# Parallel pitch processing in speech and melody: A study of the interference of musical melody on lexical pitch perception in speakers of Mandarin

**DOI:** 10.1371/journal.pone.0229109

**Published:** 2020-03-04

**Authors:** Makiko Sadakata, Joey L. Weidema, Henkjan Honing

**Affiliations:** 1 Institute for Logic, Language and Computation, Amsterdam Brain & Cognition, University of Amsterdam, Amsterdam, The Netherlands; 2 Musicology Department, University of Amsterdam, Amsterdam, The Netherlands; The Hong Kong Polytechnic University, HONG KONG

## Abstract

Music and language have long been considered two distinct cognitive faculties governed by domain-specific cognitive and neural mechanisms. Recent work into the domain-specificity of pitch processing in both domains appears to suggest pitch processing to be governed by shared neural mechanisms. The current study aimed to explore the domain-specificity of pitch processing by simultaneously presenting pitch contours in speech and music to speakers of a tonal language, and measuring behavioral response and event-related potentials (ERPs). Native speakers of Mandarin were exposed to concurrent pitch contours in melody and speech. Contours in melody emulated those in speech were either congruent or incongruent with the pitch contour of the lexical tone (i.e., rising or falling). Component magnitudes of the N2b and N400 were used as indices of lexical processing. We found that the N2b was modulated by melodic pitch; incongruent item evoked significantly stronger amplitude. There was a trend of N400 to be modulated in the same way. Interestingly, these effects were present only on rising tones. Amplitude and time-course of the N2b and N400 may suggest an interference of melodic pitch contours with both early and late stages of phonological and semantic processing.

## Introduction

Pitch is one of the most salient acoustic features shared between language and music. In language, different pitch contours can be used to denote meaning (e.g., in tonal-languages) or emphasize prosodic aspects of speech (e.g., to distinguish statements from questions or expressions). In music, pitch is a fundamental building block of what constitutes melody. Its use, however, is not nearly as dynamic or productive as it is in speech. While pitch contours in music (e.g., ascending *vs*. descending) can evoke emotive states of happiness or sadness in listeners [e.g., 1,2], their use simply does not convey meaning in the same manner as it does in language. Despite functional differences, a substantial body of research has investigated whether pitch processing in speech and music is governed by domain-specific or shared cognitive mechanisms [3–5].

There is evidence that suggests pitch to be processed by domain-specific processing mechanisms with dedicated neural resources [3,6–9]. Evidence supporting the domain-specific hypothesis originates largely from people with amusia: a pitch perception disorder characterized as an inability to steadily recognize pitch changes in music [10,11]. Recent research, however, has observed pitch discrimination deficits in both speech and music. Amusics have displayed impaired performance on distinguishing questions from statements on the basis of the final pitch glide [12]; and amusics who speak a tonal language (in which pitch is lexically productive; [13] have demonstrated significant impairment in discrimination of lexical tones [14–16]. It is now believed that the deficit affects pitch perception in both domains but is more prevalent in music as more fine-grained pitch perception is required for processing relatively small pitch intervals [10]. Processing deficits in both speech and music in amusic population suggests that pitch processing is, at least partially, governed by domain-general and shared neural mechanisms.

Additional evidence comes from studies that investigate transfer effects in professional musicians and speakers of tonal languages. Transfer describes how experience in one context can facilitate or interfere with performance in other contexts. Both behavioral and neurophysiological research focusing on transfer effects between language and music have shown that, compared to non-musicians, musicians show superior pitch perception in speech, and that speakers of tonal languages, compared to non-tonal speakers, show superior pitch perception in melody (e.g., [17–20]. Musicians and tonal speakers also tend to encode pitch more robust in the brainstem [18,21,22], and show enhanced sensory processing in cortical processing [23–32].

A more direct interference effect between the two domains has been explored by recent studies using sung stimuli. For example, when classifying speech or melodic aspect of the sung stimuli, a change in one domain significantly interferes with a speed of task performance in another domain [33]. Another study has demonstrated that neurophysiological response to auditorily rare events (Mismatch Negativity) shows no additive effects when a deviant stimulus simultaneously manipulated speech and melodic qualities, namely, vowel and pitch [34]. This finding suggests that the two domains share a detection mechanism of a change in songs at early auditory processing.

The interactions observed between language and music can be explained within a resource-sharing framework [35] in which pitch processing is proposed to be governed by shared neural resources that operate on domain-specific representations (e.g., speech categories or tonality, such as importance of tones in the tonal hierarchy). Specifically, research into the functional anatomy of pitch perception has been able to demonstrate that pitch processing is governed by shared neural substrates that display different temporal activation depending on whether sound is classified as speech or music [36,37]. Alternatively, based on the converging evidence for music-specific responses along specific neural pathways [38], it could be that brain networks that support pitch processing (and musicality in general) are partly recycled for language, thus predicting more overlap than segregation of cognitive functions [39].The current study explores the domain-specificity of pitch by investigating parallel processing of pitch contours in language and music. While numerous studies have explored transfer effects between language and music separately, i.e., from language-to-music, or from music-to-language [see e.g., 4,29], there is scarce evidence on how melodic and lexical pitch interact when processed simultaneously (see 33–35 for an example using sung speech). The current study takes a new approach to study the effect of melodic pitch on the perception of lexical pitch. We exposed native Mandarin speakers to congruent and incongruent pitch contours in speech and melody simultaneously in order to study whether they would exhibit neurophysiological sign of processing interaction. As pitch is a meaningful feature in tonal languages, we think that the response of tonal speakers can offer significant insight into its domain-specificity.

Meaningful disyllabic words in Mandarin that ended on a rising or falling lexical tone were presented simultaneously with a melody that ended on an ascending or descending gliding tone. Words were presented during the last two notes of a melody. Concurrent pitch contours in speech and melody were either congruent (i.e., glided in the same direction) or incongruent (i.e., glided in opposite directions). As indices of early and late semantic processing, event-related brain potentials (ERPs) were measured using EEG (electroencephalogram), focusing on the N2b and N400 components of the ERP. While both components are shown to be sensitive for attention, cognitive load, and conflict monitoring, these are both well-studied in the context of speech processing. Commonly, these components are associated with different stages of lexical processing, they evoke similar scalp distribution and reflect equivalent sensitivity to priming and stimulus probability [40].

The N200 is an early component with negative amplitude that peaks roughly between 180–350 ms post stimulus at frontal-central areas [41,42]. It is thought to reflect processes related to executive control, stimulus identification, novelty, and mismatch with mentally stored representations [43]. The N2-complex can be divided into smaller sub-components. The N2a, or mismatch negativity [44], is believed to reflect a (semi-) automatic encoding process elicited by a change in auditory stimuli. The N2b appears a more voluntary process and is evoked when parts of the stimulus deviate from a standard representation in memory i.e., a template mismatch [42,45]. More specifically, in speech the N2b is modulated by phonological deviations and been associated with early stages of semantic processing [42,46–48]. The N2b is often followed by the P3, a positive amplitude that peaks between 250–500 ms [49]. This response is believed to correlate to an increase of cognitive workload and reflecting stimulus probability and evaluation time.

The N400 is a negative component reaching peak amplitude around 400 ms post stimulus onset over frontal-central and central-parietal areas [50–54]. In language, the N400 component is generally believed to reflect processes related to lexical access and semantic integration. It has also been demonstrated to be involved in phonological processing [55–57]. While long believed to be a language-specific component, the N400 has been related to semantic processing in both music and language [50,58–60]. The N400 appears modulated by predictability (e.g., unpredictable or incongruent words tend to evoke larger amplitudes than predictable, expected words), and expectancy such as found in priming experiments [61].

If pitch processing is governed by shared neural mechanisms, it is expected that congruency will modulate the degree of resource allocation dedicated to concurrent pitch processing in both speech and (musical) melody and affect early and later stages of lexical access. In our study, words with incongruent pitch contours are expected to be more difficult to process than those with congruent contours and thus expected to evoke larger potentials for the N2b and N400. Contrary, if mechanisms are domain-specific, processing of concurrent incongruent melodic pitch contours will likely not affect lexical processing, and no effect would be expected on component amplitude. We analyzed the whole scalp distribution because this information likely helps us identifying components: N2b to be found in the front-central region, N400 in a wider region over scalp, and P3 in a central area.

## Method

### Ethics statement

Participants provided formal written consent prior to the start of the experiment according to the Declaration of Helsinki. The ethics committee of the Faculty of Humanities of the University of Amsterdam approved the study. All participants received a monetary fee for participation.

### Participants

Seventeen native speakers of Mandarin (10 females, mean age 25.3, *SD* = 3.6) participated in the experiment. All participants were right-handed, and reported normal or corrected vision and normal hearing (self-report). None reported any known neurological impairment. All participants were non-musicians with no form of musical training in at least the last 5 years.

### Materials

The materials comprised both short melodies and disyllabic words in Mandarin. The speech material consisted of 36 meaningful tonal minimal pairs in Mandarin with rising (tone 2) and falling (tone 4) lexical tones (e.g., yu2 lei2 [鱼雷: ‘torpedo’] *vs*. yu2 lei4 [鱼类: ‘fish’]). Words were all nouns and differed only in meaning by the direction of the lexical tone on the last syllable. All minimal pairs were matched on lexical frequency (all *p-*values > .24) as reported in the SUBTLEX-CH database [62]. Stimuli were read out loud at a constant rate by a female native speaker of Mandarin and recorded at a sampling rate of 44.1 kHz. All syllables were time normalized to 500 ms using custom code in PRAAT [63]. No other modifications were done to the pitch contour.

Melodic materials consisted of four melodies (approximately 8 notes in length). Melodies were arranged in a diatonic scale with a total length of 3,000 ms (one quarter note equaled 500 ms). The last quarter note position in the melody always contained an ascending or descending glissando (i.e., analogous to the lexical contour of the critical item in speech). All melodies were synthesized to MIDI flute using *FluidSynth* sound font (http://www.fluidsynth.org/) and custom written code in Supercollider (http://supercollider.github.io/).

Speech stimuli were superimposed on the two last quarter notes positions of a melody (i.e., during the last 1,000 ms) and merged in a single mono signal. Prior to this, both speech and melodic items were normalized in terms of loudness. The materials comprised of congruent and incongruent test items. On congruent items, the direction of the lexical tone matched the direction of the last melodic contour (items with rising speech contour [C^R^] and items with falling speech contour [C^F^]). On incongruent items, the direction of pitch in speech diverged from pitch in melody (items with rising speech contour [I^R^] and items with falling speech contours [I^F^]).

**Fig 1:**
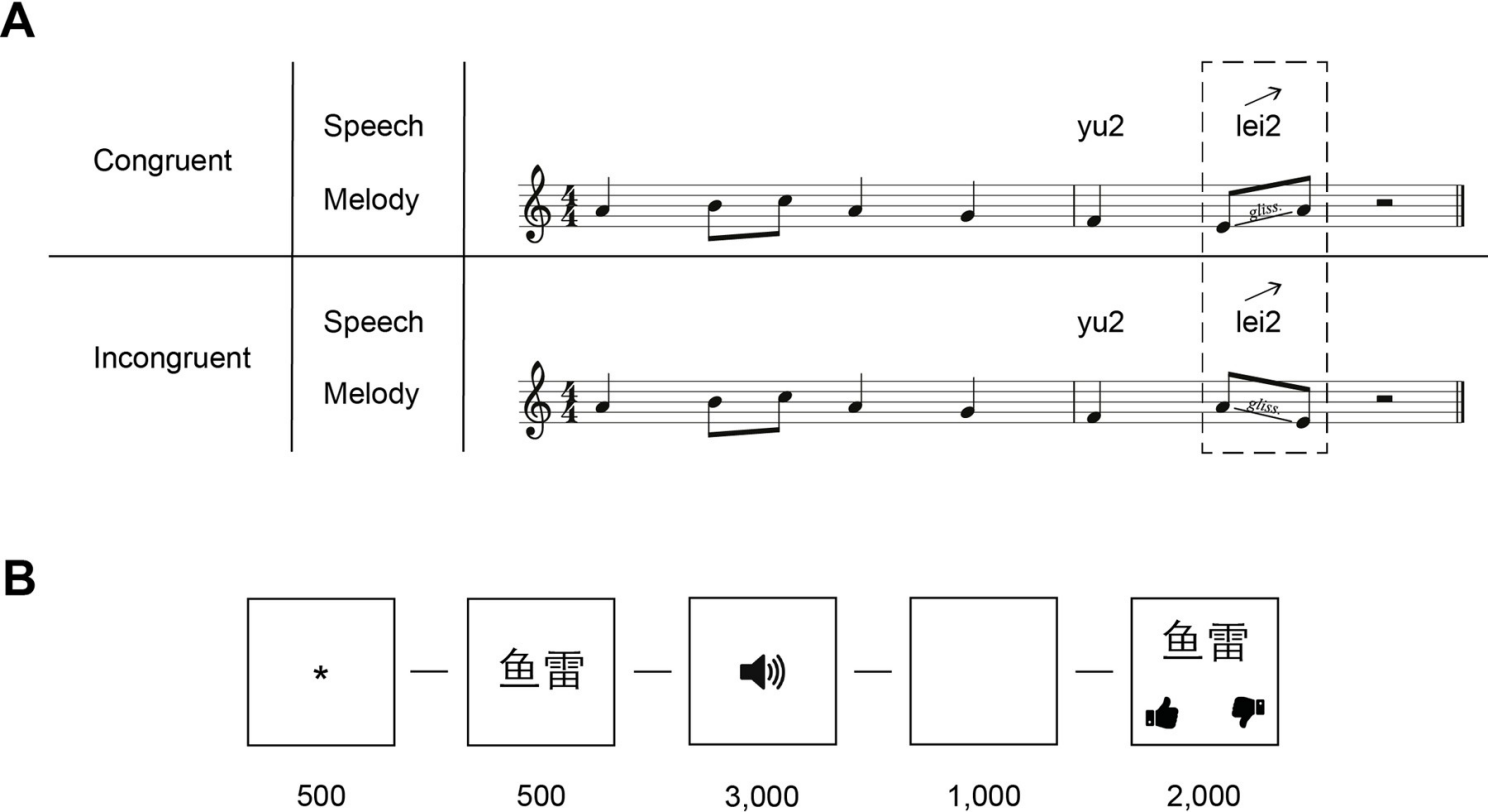
Examples of stimuli and the order of events for each experimental trial. **(A)** provides an example of stimuli with a rising lexical tone (C^R^ and I^R^). Each row contains speech (written out in pinyin) and the music notation. The dashed rectangle highlights the critical item. The arrow above the last syllable indicates the direction of the lexical tone. In **(B)** the order of events for each trial with time shown in milliseconds (see *Procedure* below).

The complete set of materials comprised a total of 144 items: 72 were congruent (36 C^R^, 36 C^F^) and 72 were incongruent (36 I^R^, 36 I^F^). In order to prime lexical processing, each trial was preceded by a visual cue on screen: a Mandarin character that corresponded to the word that followed. Table 1 contains an overview of the experimental items and the abbreviations used. Fig 1A contains an example of a stimulus (C^R^ and I^R^). Example audio stimuli can be found in S1 Audio.

**Table 1:** A schematic overview of the pitch contours of congruent and incongruent experimental items (and their abbreviations).

Domain	Congruent		Incongruent	
**Speech**	Rising	Falling	Rising	Falling
**Melody**	Rising	Falling	Falling	Rising
*Abbr*.	C^R^	C^F^	I^R^	I^F^

### Procedure

Participants sat in front of a screen in a soundproof room and were asked to move as little as possible. They were told they would listen to a series of melodies with words embedded in them. Participants had to pay attention to the word and listen to its meaning. Participants were informed that after each item, they would perform a lexical verification task. In this task, a Mandarin character (the rising or falling member of the minimal pair) would appear on screen and participants had to answer (yes/no) whether this character corresponded to the word they heard by pressing a key on a keyboard.

The onset of each experimental trial was prompted with an asterisk on screen for 500 ms. A Mandarin character (the prime) then appeared on screen for 500 ms. The screen then went blank and an experimental item was presented through speakers (3,000 ms). A blank screen for 1,000 ms then preceded a timed two-alternative forced choice lexical verification question for 2,000 ms—this time constraint was implemented to ensure rapid processing and control for attention as much as possible. The end of a trial was marked by a blank screen for 1,000 ms. Fig 1B displays a graphical representation of the order of events of an experimental trial.

Stimuli were pseudo-randomized with the sole restriction that an item could not be presented twice in a row. Stimuli were presented in 9 blocks of 48 items (432 trials in total) at approximately 70 dB. Each experimental item was thus presented three times. Participants performed a practice session prior to the start of the experiment (with 12 items not used in the experiment) and were given feedback when appropriate. Behavioral response (percentages correct) and ERPs were recorded. The entire experiment lasted approximately 120 minutes (including self-paced breaks between blocks).

### EEG acquisition

The EEG was recorded continuously from 64 scalp locations using Ag–AgCl electrodes (international 10/20 system). Eye blinks and other ocular artifacts were recorded with a bipolar montage attached to the exterior canthi and the infraorbital and supraorbital regions of the right eye. Two additional electrodes were placed at the left and right mastoids. A Common Mode Sense (CMS) and Driven Right Leg (DRL) electrode were used as reference. The signal was amplified using a Biosemi ActiveTwo AD-box (Biosemi, Amsterdam, The Netherlands) with a band-pass of 0.1–100 Hz. Electrode impedance threshold was kept below 5 kΩ. The signal was digitized at a sample rate of 8 kHz with a 16-bit resolution.

### EEG pre-processing

The data were down-sampled to 512 Hz, re-referenced offline to the algebraic mean of the left and right mastoids and band-pass filtered (infinite impulse response [IIR], 0.1–30 Hz, 24 dB/octave). Data for all participants were visually inspected for bad channels and significant line drift–these segments were manually removed. Eye blinks were removed by subjecting the data to independent component analysis, and bad channels were interpolated with the mean value of its closest neighbors (3–4 electrodes). The data for each condition (C^R^, C^F^, I^R^, I^F^) were time locked to the last syllable (critical item) and segmented into separate epochs from -150 ms pre-stimulus onset to 1,000 post-stimulus (baseline corrected relative to stimulus onset). Epochs that contained amplitude variation exceeding 150 μV in a 500 ms sliding window (step size 250 ms) were rejected. The 5.61% of the trials were rejected on average. One participant with a rejection rate higher than 25% was excluded from analyses [64]. Pre-processing was conducted using custom code for MATLAB (vR2013b, www.mathworks.com) and the EEGLAB (v13.5.4b) [65] and ERPLAB (v6.1.2) [66] toolboxes.

### Statistical analyses

The waveforms revealed distinct ERP modulations around 200, 300 and 400 ms but latency and component magnitude between both levels of Direction and Congruency differed substantially. Peak latencies for the N2b and N400 were measured at electrode Afz where potentials reached maxima. There were differences in observed latency peaks between conditions in the N2b latency: C^R^ (205 ms), C^F^ (176 ms), I^R^ (215 ms), I^F^ (168 ms). The N2b was subsequently defined as the mean amplitude in time interval 150–225 ms. A positive deflection around 300 ms for C^F^ and I^F^ reached maximum at 334 ms and 260 ms, respectively. This possible P3 component was quantified as the mean amplitude between 250–360 ms. We found no apparent P3 in the rising contour conditions. Latency peaks for the N400 latency illustrated differences between Direction and Congruency, and only C^R^ (441 ms) and I^R^ (445 ms) evoked negative peaks. The N400 was defined as the mean amplitude between 400–500 ms. These ranges correspond with that of previously studied N2b, P3 and N400 peaks using musical stimuli [e.g., 67,68].

**Fig 2:**
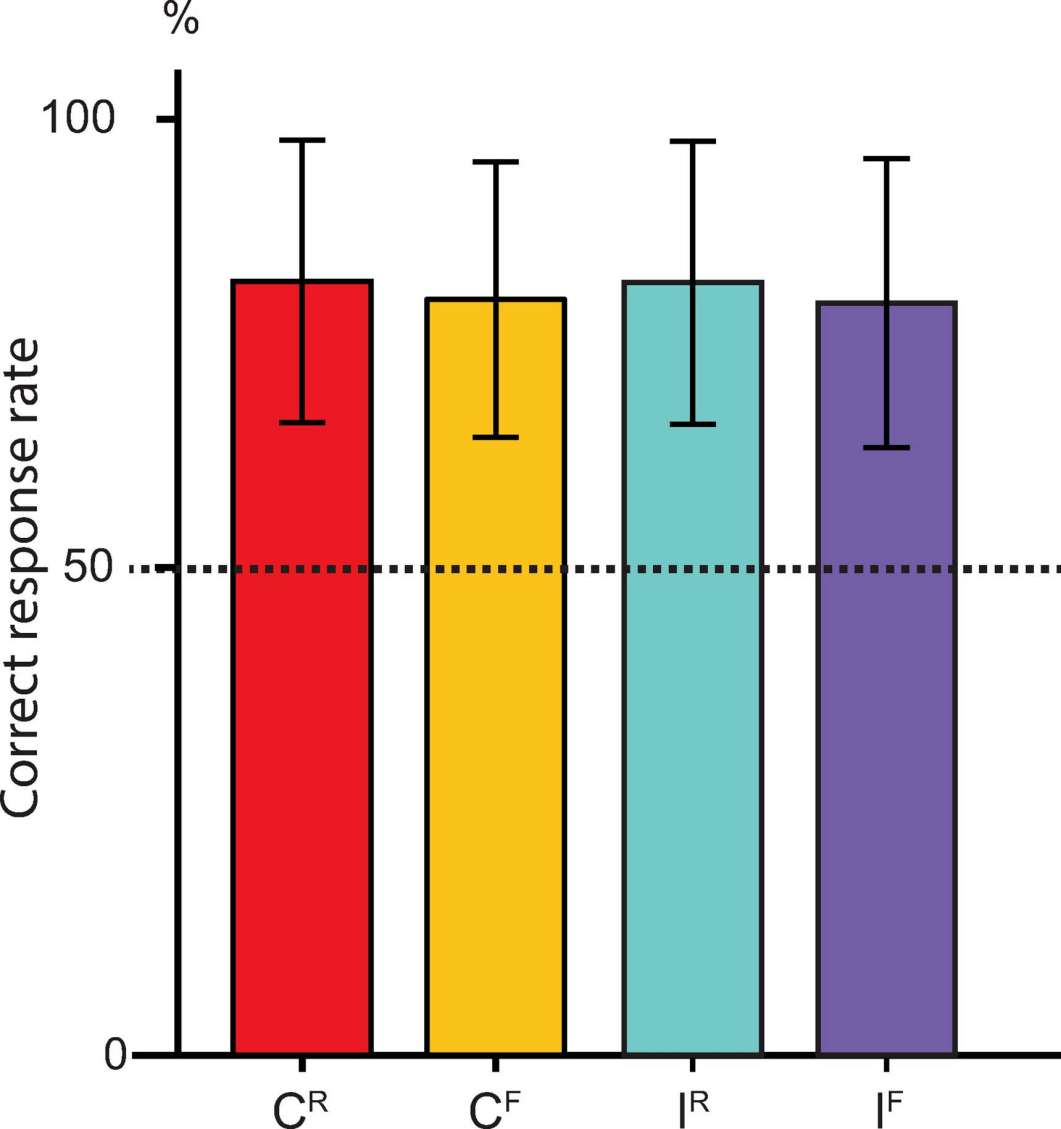
Correct response rate for the lexical verification task. Behavioral scores are displayed in percentages (errors bars indicate 95% coincidence intervals). The dashed line indicates chance level. C^R^ (rising speech with rising melodic contour), C^F^ (falling speech with falling melodic contour), I^R^ (rising speech with falling melodic contour), I^F^ (falling speech with rising melodic contour).

Mean amplitude of each component (defined as the average amplitude in each latency window), topography and morphology modulated by congruency of contour (congruent *vs*. incongruent), was investigated using repeated-measures analyses on nine regions of interest (see S1 Fig). A perceptual asymmetry between rising and falling pitch contours for speakers of Mandarin [69–71] motivated us to analyze speech items with rising and falling lexical tone separately. Analyses were thus parameterized with Congruency (congruent *vs*. incongruent) as main effect, and Caudality (anterior, central, posterior) and Laterality (left, right, midline) as factors for each rising and falling contours. We applied Bonferroni correction to cover 5*3-way ANOVAs in this experiment (cutoff = 0.05/35, p<0.0014) when interpreting main effects. In case of discussing marginally significant effects, we considered effect size as a supporting factor. In case of violation of sphericity, Greenhouse-Geiser *p*-values are reported with *Ɛ*. As an estimate of effect size, partial eta-squared ($\eta_{p}^{2}$) is reported. Bonferroni corrected *p*-values were used for all post hoc pairwise comparisons. Fig 2 shows the grand average ERP waveforms and scalp distribution maps for each analysis window.

## Results

**Fig 3:**
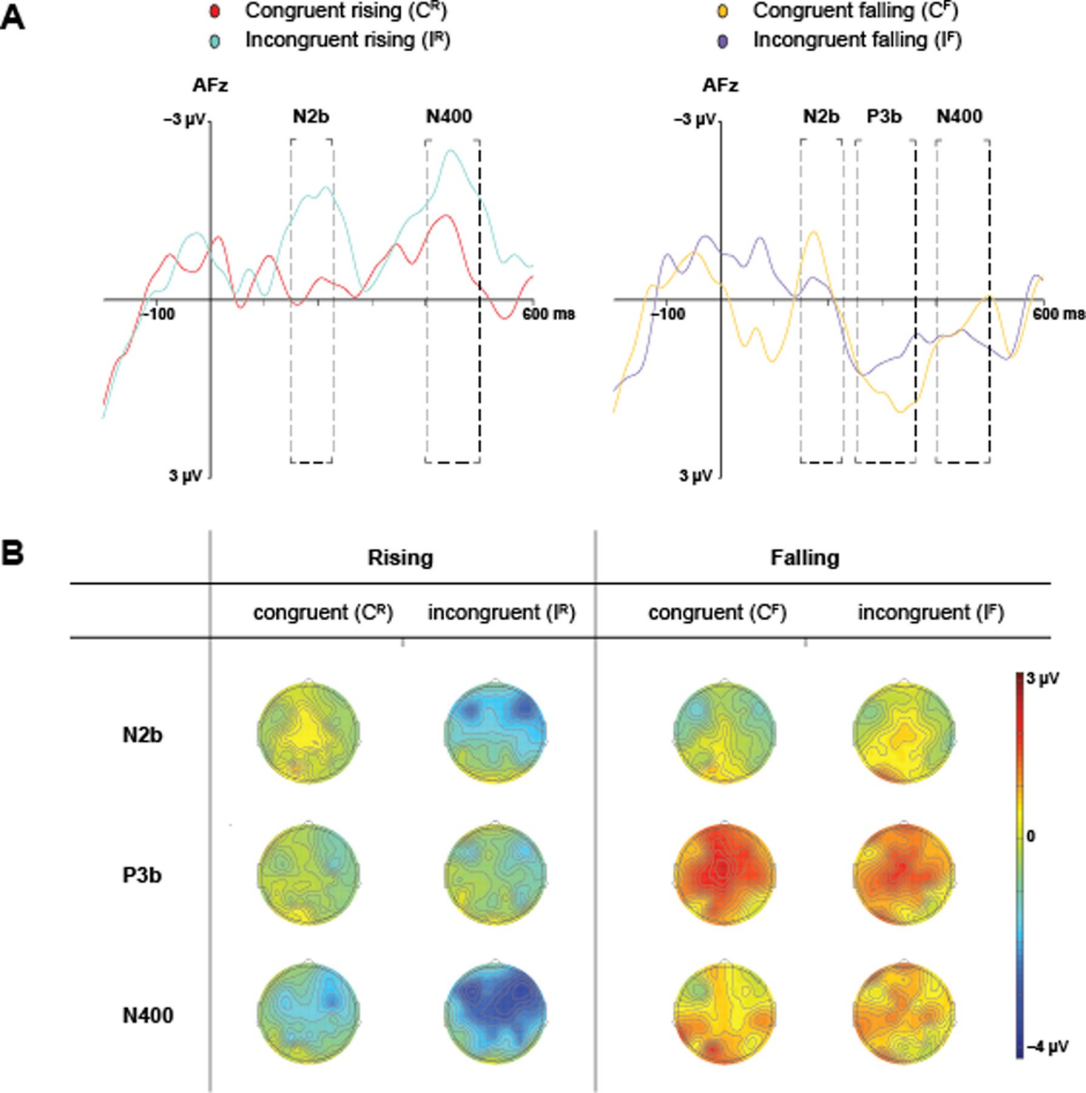
Grand average waveforms and scalp maps. **(A)** shows the grand average ERP waveform for all participants at electrode AFz. The left graph contrasts the waveforms for congruent and incongruent items with rising speech contours. The right graph contrasts that with falling contours. Dashed boxes demarcate latency windows used in the statistical analyses for the N2b (150–225 ms), P3b (250–360 ms) and N400 (400–500 ms). The y-axes denote amplitude in microvolts (-3 μV to 3 μV). The x-axes time in milliseconds (-200 ms to 600 ms). **(B)** contains topographical maps that display the scalp distribution averaged over the latency windows used in the analyses.

Behavioral scores from the lexical verification task were higher than chance and statistically indistinguishable for rising and falling tones in both congruent and incongruent conditions (approximately 82% correct in all conditions, see Fig 2). The effects for the N2b and N400 were found to be strongest over anterior and central locations of both hemispheres which appears consistent with patterns evoked by words presented in isolation for English, Spanish and Mandarin listeners [e.g.,^36^,^47^,^48^,^66^,^69^, see Fig 3].

### Rising speech contours

In the N2b latency, there were main effects of Congruency [*F*(1,15) = 20.94, *p* < .001, $\eta_{p}^{2}$ = .583] with significant interaction with Caudality [*F*(1.23,18.50) 8.65, *p* = .006 (unadjusted *p* = .001: Greenhouse-Geiser *Ɛ* = .62), $\eta_{p}^{2}$ = .366]. The main effect of Caudality and Lateralization did not reach significance (Caudality [*F*(1.24,18.58) = 6.75, *p* = .013 (unadjusted *p* = .004: Greenhouse-Geiser *Ɛ* = .62), $\eta_{p}^{2}$ = .310], Lateralization [*F*(2,30) = 2.81, *p* = .076]) nor other interactions. Post hoc pairwise comparison indicated the difference between congruent and incongruent conditions to be significant at anterior (mean difference 1.856 μV, *p* < .001) and central (mean difference 1.475 μV, *p* < .001) sites. The analysis thus indicated significantly greater negative deflections at anterior and central sites for incongruent condition than for congruent condition.

In the N400 latency, there was a tendency of all three main effects to be significant (Congruency [*F*(1,15) = 5.86, *p* = .029, $\eta_{p}^{2}$ = .281], Caudality [*F*(2,30) = 7.28, *p* = .003, $\eta_{p}^{2}$ = .327], Laterality [*F*(2,30) = 3.33, *p* = .049, $\eta_{p}^{2}$ = .182]) with a interaction of Caudality and Laterality [*F*(2, 30) = 2.66, *p* = .041, $\eta_{p}^{2}$ = .151]. Although their significance fell short due to our very conservative correction for p-values, the analysis indicated reliable effect sizes for the main effects of Congruency and Caudality, potentially indicating that I^R^ evoked overall stronger negative deflections than C^R^.

### Falling speech contours

The N2b analysis did not indicate any significant main effects.

The P3 analysis indicated strong main effect of Lateraity [*F*(2,30) = 10.27, *p* < .001, $\eta_{p}^{2}$ = .406]. Posthoc comparison indicated central sites were significantly more positive than the right sites. No other effects were significant.

In the N400 window, none of the main effects were significant.

## Discussion

An extensive body of research has shown that experience with pitch in one domain can influence pitch perception in another. While the literature on transfer of pitch abilities between language and music is abundant, there are few studies that have explored simultaneous processing of pitch in both domains. The current study explored parallel processing of congruent and incongruent pitch contours in language and music. Native speakers of Mandarin were exposed to congruent and incongruent pitch contours in melody and speech. Results showed that processing two concurrent pitch contours did not affect lexical verification at a behavioral level. However, at a neurophysiological level, significant N2b response and a marginally significant N400 response were observed when incongruent pitch contours were presented. Although it was rathe unexpected that this effect only occurred in the rising contour conditions (see the discussion below), we propose that this can be taken as an indication that melodic pitch processing interacting with early and late stages of lexical processing: incongruent pitch contours would interfere with lexical processing and would elicit greater negative deflections for the N2b and N400.

It is interesting that speech with only falling lexical tones evoked a positive component (P3b). Importantly, the amplitude of this component was not modulated by our Congruency manipulation, therefore, we think that this is caused by a more general feature of falling stimuli. In spoken Mandarin, a clear durational contrast exists between words with rising and falling tones. Previous behavioral studies have been able to demonstrate differences in the perception of both tones in language and music for speakers of Mandarin as a result of top-down interference from language experience [69–71]. In the current study, syllable duration was time equalized between rising and falling tones and a probable explanation for the P3b might thus lie in the longer duration of the falling tone. As a falling lexical tone in Mandarin is characterized by a rather short duration and steep slope, speech with falling tones could have been perceived as artificial or physically deviant. We think that the P3b evoked in the current study reflects the recruitment of additional cognitive resources needed to processes the deviant pitch contour in this condition. Furthermore, the positive amplitude may have subdued an observable N400 effect. In line with this argument, congruent items in falling items evoked larger P3b than incongruent items, although not significant.

While we believe that our study opens up interesting future directions, because of the exploratory nature, our findings should be interpreted with care. One may argue that the domain-specific mechanism would still predict enhanced component amplitude because both domains would simultaneously activate similar neural mechanisms for semantic information processing. Here, we argue that, in our experiment, it is likely that top-down effects from language will prioritize the processing of lexical pitch over pitch in melody because, while our participants were primed to expect lexical tones to go up or down, such expectation was absent for concurrent musical pitch (the melodies could end naturally in both up and down).

Another important issue is the fact that the N2b and N400 are sensitive to other processes than the pure linguistic processing. For example, the level of attention required to perform a task is known to modulate the amplitude of both components [72]. In this light, it is possible that the incongruent condition may have been more attention demanding than the congruent condition in both experiments, which led participants to produce enhanced N2b and N400 responses. Adding an extra control condition with noise or unrelated environment sounds combined with lexical tone stimuli would be an excellent control to isolate the responses related to musical and linguistic processing.

Future studies could also include a task focusing on pitch processing in both speech and melody. To further explore the interaction between both domains, it would be interesting to investigate the role of speech-specific modulation from language on tonal perception by exposing non-tonal speakers to concurrent pitch contours. As non-tonal speakers will have no knowledge of Mandarin semantics, bottom-up acoustic signal processing can be directly compared to the top-down listening strategies of the Mandarin speakers. By differentiating between bottom-up and top-down processing of pitch contours in both domains, we can significantly broaden our understanding of the domain-specificity of pitch cognition and pitch perception in language and music.

To sum up, we used an explorative approach to address cross-domain interaction effects between melodic and lexical pitch processing mechanisms in speakers of Mandarin. Congruency between concurrent melodic and lexical pitch contours seemed to affect phonological and semantic processing at the neurophysiological but not the cognitive level. Since no observable behavioral effect of melodic pitch on lexical verification was found, interaction between domains might be restricted to lower level acoustic processing. This asymmetry between cognition and neurophysiology has been reported in other studies concerning pitch processing in speech and music [see 21,22]. Our findings are in agreement with cross-domain interaction that processing concurrent incongruent pitch contours increases semantic processing load. Such cross-domain interaction suggests melodic and lexical pitch processing to rely on shared processing mechanisms that compete for cognitive resources during early and late stages of phonological and semantic processing. However, the asymmetric results between the rising and falling items reveal that this interaction is susceptible to acoustic properties of the stimuli as well as the listening experience of the participants.

## Supporting information

S1 Fig Figure of regions of interest used in the analyses of ERPs in experiment. Regions are divided from top to bottom by Caudality (anterior, central, posterior) and from left to right by Laterality (left, midline, right).
(PNG)

S1 Data The data used in the statistical analyses of component latency windows. (ZIP)

S1 Audio Auditory stimuli examples. **A:** Both speech and melodic contours rising (C^R^). **B:** I^R^ Rising speech contour presented with falling melodic contour.
(WAV)

## References

[1] Gerardi GM, Gerken L. The Development of Affective Responses to Modality and Melodic Contour. Music Percept. 1995;

[2] Gagnon L, Peretz I. Mode and tempo relative contributions to “happy-sad” judgements in equitone melodies. Cognition and Emotion. 2003. 10.1080/02699930302279 29715736

[3] Peretz I, Vuvan D, Lagrois MÉ, Armony JL. Neural overlap in processing music and speech. Philosophical Transactions of the Royal Society B: Biological Sciences. 2015. 10.1098/rstb.2014.0090 PMC4321131 25646513

[4] Asaridou SS, McQueen JM. Speech and music shape the listening brain: Evidence for shared domain-general mechanisms. Front Psychol. 2013; 10.3389/fpsyg.2013.00321 PMC3671174 23761776

[5] Patel AD. Sharing and Nonsharing of Brain Resources for Language and Music. In: Language, Music, and the Brain. 2015.

[6] Peretz I. Music, language and modularity framed in action. Psychol Belg. 2009;

[7] Zatorre RJ, Baum SR. Musical melody and speech intonation: Singing a different tune. PLoS Biol. 2012;10(7):5. 10.1371/journal.pbio.1001372 PMC3409119 22859909

[8] Tierney A, Dick F, Deutsch D, Sereno M. Speech versus song: Multiple pitch-sensitive areas revealed by a naturally occurring musical illusion. Cereb Cortex. 2013;23(2):249–54. 10.1093/cercor/bhs003 22314043 PMC3539450

[9] Norman-Haignere S, Kanwisher NG, McDermott JH. Distinct Cortical Pathways for Music and Speech Revealed by Hypothesis-Free Voxel Decomposition. Neuron. 2015; 10.1016/j.neuron.2015.11.035 PMC4740977 26687225

[10] Peretz I, Hyde KL. What is specific to music processing? Insights from congenital amusia. Trends Cogn Sci. 2003 8;7(8):362–7. 10.1016/s1364-6613(03)00150-5 12907232

[11] Peretz I. Neurobiology of Congenital Amusia. Trends in Cognitive Sciences. 2016. 10.1016/j.tics.2016.09.002 27692992

[12] Patel AD, Wong M, Foxton J, Lochy A, Peretz I. Speech intonation perception deficits in musical tone deafness (congenital amusia). Music Percept. 2008; 10.1525/MP.2008.25.4.315

[13] Yip M. Tone. Cambridge: Cambridge University Press; 2002.

[14] Nguyen S, Tillmann B, Gosselin N, Peretz I. Tonal language processing in congenital amusia. In: Annals of the New York Academy of Sciences 2009. 10.1111/j.1749-6632.2009.04855.x 19673828

[15] Tillmann B, Rusconi E, Traube C, Butterworth B, Umiltà C, Peretz I. Fine-grained pitch processing of music and speech in congenital amusia. J Acoust Soc Am. 2011; 10.1121/1.3658447 22225063

[16] Tillmann B, Burnham D, Nguyen S, Grimault N, Gosselin N, Peretz I. Congenital amusia (or tone-deafness) interferes with pitch processing in tone languages. Front Psychol. 2011; 10.3389/fpsyg.2011.00120 PMC3119887 21734894

[17] Bidelman GM, Hutka S, Moreno S. Tone Language Speakers and Musicians Share Enhanced Perceptual and Cognitive Abilities for Musical Pitch: Evidence for Bidirectionality between the Domains of Language and Music. PLoS One. 2013; 10.1371/journal.pone.0060676 PMC3614545 23565267

[18] Bidelman GM, Gandour JT, Krishnan A. Musicians and tone-language speakers share enhanced brainstem encoding but not perceptual benefits for musical pitch. Brain Cogn. 2011; 10.1016/j.bandc.2011.07.006 PMC3159732 21835531

[19] Delogu F, Lampis G, Belardinelli MO. From melody to lexical tone: Musical ability enhances specific aspects of foreign language perception. Eur J Cogn Psychol. 2010; 10.1080/09541440903155658

[20] Marie C, Delogu F, Lampis G, Belardinelli MO, Besson M. Influence of musical expertise on segmental and tonal processing in Mandarin Chinese. J Cogn Neurosci. 2011; 10.1162/jocn.2010.21585 20946053

[21] Wong PCM, Skoe E, Russo NM, Dees T, Kraus N. Musical experience shapes human brainstem encoding of linguistic pitch patterns. Nat Neurosci [Internet]. 2007 4 [cited 2016 Sep 22];10(4):420–2. Available from: http://www.ncbi.nlm.nih.gov/pubmed/17351633 doi: 10.1038/nn1872 17351633 10.1038/nn1872 PMC4508274

[22] Bidelman GM, Gandour JT, Krishnan A. Musicians demonstrate experience-dependent brainstem enhancement of musical scale features within continuously gliding pitch. Neurosci Lett. 2011; 10.1016/j.neulet.2011.08.036 PMC3196385 21906656

[23] Chandrasekaran B, Krishnan A, Gandour JT. Relative influence of musical and linguistic experience on early cortical processing of pitch contours. Brain Lang. 2009; 10.1016/j.bandl.2008.02.001 PMC2670545 18343493

[24] Hutka S, Bidelman GM, Moreno S. Pitch expertise is not created equal: Cross-domain effects of musicianship and tone language experience on neural and behavioural discrimination of speech and music. Neuropsychologia. 2015; 10.1016/j.neuropsychologia.2015.03.019 25797590

[25] Xu Y, Krishnan A, Gandour JT. Specificity of experience-dependent pitch representation in the brainstem. Neuroreport. 2006; 10.1097/01.wnr.0000236865.31705.3a 17001276

[26] Chandrasekaran B, Gandour JT, Krishnan A. Neuroplasticity in the processing of pitch dimensions: A multidimensional scaling analysis of the mismatch negativity. Restor Neurol Neurosci. 2007; PMC4380289 17942999

[27] Krishnan A, Gandour JT, Bidelman GM, Swaminathan J. Experience-dependent neural representation of dynamic pitch in the brainstem. Neuroreport. 2009; 10.1097/WNR.0b013e3283263000 PMC2692950 19223793

[28] Baumann S, Meyer M, Jäncke L. Enhancement of auditory-evoked potentials in musicians reflects an influence of expertise but not selective attention. J Cogn Neurosci. 2008; 10.1162/jocn.2008.20157 18457513

[29] Oechslin MS. The plasticity of the superior longitudinal fasciculus as a function of musical expertise: a diffusion tensor imaging study. Front Hum Neurosci. 2010;3(February):1–12. 10.3389/neuro.09.076.2009 PMC2821183 20161812

[30] Ott CGM, Langer N, Oechslin MS, Meyer M, Jäncke L. Processing of voiced and unvoiced acoustic stimuli in musicians. Front Psychol. 2011; 10.3389/fpsyg.2011.00195 PMC3167375 21922011

[31] Kühnis J, Elmer S, Meyer M, Jäncke L. The encoding of vowels and temporal speech cues in the auditory cortex of professional musicians: An EEG study. Neuropsychologia. 2013; 10.1016/j.neuropsychologia.2013.04.007 23664833

[32] Oechslin MS, Meyer M, Jäncke L. Absolute pitch-functional evidence of speech-relevant auditory acuity. Cereb Cortex. 2010; 10.1093/cercor/bhp113 PMC2803739 19592570

[33] Kolinsky R, Lidji P, Peretz I, Besson M, Morais J. Processing interactions between phonology and melody: Vowels sing but consonants speak. Cognition. 2009; 10.1016/j.cognition.2009.02.014 19409537

[34] Lidji P, Jolicœur P, Kolinsky R, Moreau P, Connolly JF, Peretz I. Early integration of vowel and pitch processing: A mismatch negativity study. Clin Neurophysiol. 2010; 10.1016/j.clinph.2009.12.018 20071227

[35] Patel AD. Language, music, and the brain: A resource-sharing framework. In: Language and Music as Cognitive Systems. 2012.

[36] Abrams DA, Bhatara A, Ryali S, Balaban E, Levitin DJ, Menon V. Decoding temporal structure in music and speech relies on shared brain resources but elicits different fine-scale spatial patterns. Cereb Cortex. 2011; 10.1093/cercor/bhq198 PMC3116734 21071617

[37] Rogalsky C, Rong F, Saberi K, Hickok G. Functional anatomy of language and music perception: Temporal and structural factors investigated using functional magnetic resonance imaging. J Neurosci. 2011; 10.1523/JNEUROSCI.4515-10.2011 PMC3066175 21389239

[38] Peretz I, Vuvan D, Armony JL. Neural overlap in processing music and speech. Phil Trans R Soc B 370 20140090. 2015; 10.1098/rstb.2014.0090 PMC4321131 25646513

[39] Honing H. Musicality as an Upbeat to Music: Introduction and Research Agenda. In: The Origins of Musicality. 2019.

[40] Deacon D, Breton F, Ritter W, Vaughan HG. The relationship between N2 and N400: Scalp distribution, stimulus probability, and task relevance. Psychophysiology. 1991;28(2):185–200. 10.1111/j.1469-8986.1991.tb00411.x 1946885

[41] Sutton S, Braren M, Zubin J, John ER. Evoked-potential correlates of stimulus uncertainty. Science (80-). 1965; 10.1126/science.150.3700.1187 5852977

[42] Patel SH, Azzam PN. Characterization of N200 and P300: Selected studies of the Event-Related Potential. International Journal of Medical Sciences. 2005. 10.7150/ijms.2.147 PMC1252727 16239953

[43] Folstein JR, Van Petten C. Influence of cognitive control and mismatch on the N2 component of the ERP: A review. Psychophysiology. 2008. 10.1111/j.1469-8986.2007.00602.x PMC2365910 17850238

[44] Näätänen R, Paavilainen P, Rinne T, Alho K. The mismatch negativity (MMN) in basic research of central auditory processing: A review. Clinical Neurophysiology. 2007. 10.1016/j.clinph.2007.04.026 17931964

[45] Sams M, Alho K, Näätänen R. Sequential effects on the ERP in discriminating two stimuli. Biol Psychol. 1983; 10.1016/0301-0511(83)90065-0 6626636

[46] Schmitt BM, Kutas M, Münte TF. Electrophysiological estimates of the time course of semantic and phonological encoding during implicit picture naming. Neuropsychologia. 2000;37(September 2000):473–84. 10934906

[47] Van Den Brink D, Brown CM, Hagoort P. Electrophysiological evidence for early contextual influences during spoken-word recognition: N200 versus N400 effects. J Cogn Neurosci. 2001; 10.1162/089892901753165872 11595099

[48] D’Arcy RCN, Connolly JF, Crocker SF. Latency shifts in the N2b component track phonological deviations in spoken words. Clin Neurophysiol. 2000; 10.1016/s1388-2457(99)00210-2 10656509

[49] Polich J. Updating P300: An integrative theory of P3a and P3b. Clinical Neurophysiology. 2007. 10.1016/j.clinph.2007.04.019 PMC2715154 17573239

[50] Kutas M, Hillyard SA. Brain potentials during reading reflect word expectancy and semantic association. Nature. 1984; 10.1038/307161a0 6690995

[51] Kutas M, Hillyard SA. Reading senseless sentences: brain potentials reflect semantic incongruity. Vol. 207, Science (New York, N.Y.). 1980 p. 203–5. 10.1126/science.7350657 7350657

[52] Schirmer A, Tang S-L, Penney TB, Gunter TC, Chen H-C. Brain responses to segmentally and tonally induced semantic violations in Cantonese. J Cogn Neurosci. 2005 1;17(1):1–12. 10.1162/0898929052880057 15701235

[53] Huang X, Yang JC, Chang R, Guo C. Task modulation of disyllabic spoken word recognition in Mandarin Chinese: A unimodal ERP study. Sci Rep. 2016; 10.1038/srep25916 PMC4867628 27180951

[54] Huang X, Yang J-C, Zhang Q, Guo C. The time course of spoken word recognition in Mandarin Chinese: A unimodal ERP study. Neuropsychologia. 2014;63(May):165–74. 25172388 10.1016/j.neuropsychologia.2014.08.015

[55] Dumay N, Benraïss A, Barriol B, Colin C, Radeau M, Besson M. Behavioral and electrophysiological study of phonological priming between bisyllabic spoken words. J Cogn Neurosci. 2001; 10.1162/089892901564117 11224913

[56] Praamstra P, Meyer AS, Levelt WJM. Neurophysiological manifestations of phonological processing: Latency variation of a negative ERP component timelocked to phonological mismatch. J Cogn Neurosci. 1994; 10.1162/jocn.1994.6.3.204 23964972

[57] Perrin F, García-Larrea L. Modulation of the N400 potential during auditory phonological/semantic interaction. Cogn Brain Res. 2003; 10.1016/s0926-6410(03)00078-8 12763190

[58] Koelsch S, Kasper E, Sammler D, Schulze K, Gunter T, Friederici AD. Music, language and meaning: Brain signatures of semantic processing. Nat Neurosci. 2004; 10.1038/nn1197 14983184

[59] Nieuwland MS, Van Berkum JJA. When peanuts fall in love: N400 evidence for the power of discourse. J Cogn Neurosci. 2006; 10.1162/jocn.2006.18.7.1098 16839284

[60] Steinbeis N, Koelsch S. Comparing the processing of music and language meaning using EEG and fMRI provides evidence for similar and distinct neural representations. PLoS One. 2008;3(5):1–7. 10.1371/journal.pone.0002226 PMC2376093 18493611

[61] Kutas M, Federmeier KD. Thirty Years and Counting: Finding Meaning in the N400 Component of the Event-Related Brain Potential (ERP). Annu Rev Psychol. 2011; 10.1146/annurev.psych.093008.131123 PMC4052444 20809790

[62] Cai Q, Brysbaert M. SUBTLEX-CH: Chinese word and character frequencies based on film subtitles. PLoS One. 2010; 10.1371/journal.pone.0010729 PMC2880003 20532192

[63] Boersma P, Weenink D. Praat: doing phonetics by computer (version 5.3.84). 2014.

[64] Luck SJ. An introduction to the event-related potential technique. Cambridge, MA: MIT Press; 2005.

[65] Delorme A, Makeig S. EEGLAB: An open source toolbox for analysis of single-trial EEG dynamics including independent component analysis. J Neurosci Methods. 2004; 10.1016/j.jneumeth.2003.10.009 15102499

[66] Lopez-Calderon J, Luck SJ. ERPLAB: An open-source toolbox for the analysis of event-related potentials. Front Hum Neurosci. 2014; 10.3389/fnhum.2014.00213 PMC3995046 24782741

[67] Tervaniemi M, Kruck S, De Baene W, Schröger E, Alter K, Friederici AD. Top-down modulation of auditory processing: Effects of sound context, musical expertise and attentional focus. Eur J Neurosci. 2009; 10.1111/j.1460-9568.2009.06955.x 19821835

[68] Koelsch S, Gunter TC, Wittfoth M, Sammler D. Interaction between syntax processing in language and in music: An ERP study. J Cogn Neurosci. 2005; 10.1162/089892905774597290 16269097

[69] Peretz I, Nguyen S, Cummings S. Tone language fluency impairs pitch discrimination. Front Psychol. 2011; 10.3389/fpsyg.2011.00145 PMC3131151 21772825

[70] Bent T, Bradlow AR, Wright BA. The influence of linguistic experience on the cognitive processing of pitch in speech and nonspeech sounds. J Exp Psychol Hum Percept Perform. 2006; 10.1037/0096-1523.32.1.97 16478329

[71] Weidema JL, Roncaglia-Denissen MP, Honing H. Top-Down modulation on the Perception and categorization of identical pitch contours in speech and music. Front Psychol. 2016; 10.3389/fpsyg.2016.00817 PMC4889578 27313552

[72] Erlbeck H, Kübler A, Kotchoubey B, Veser S. Task instructions modulate the attentional mode affecting the auditory MMN and the semantic N400. Front Hum Neurosci. 2014; 10.3389/fnhum.2014.00654 PMC4145469 25221494

