# Correction: Parallel pitch processing in speech and melody: A study of the interference of musical melody on lexical pitch perception in speakers of Mandarin

**DOI:** 10.1371/journal.pone.0283262

**Published:** 2023-03-13

**Authors:** Makiko Sadakata, Joey L. Weidema, M. Paula M. Roncaglia-Denissen, Henkjan Honing

Paula M. Roncaglia-Denissen should be included in the author byline instead of the Acknowledgments. Paula M. Roncaglia-Denissen should be listed as the third author, and their affiliation is 3: Department of Cognitive Science and Artificial Intelligence, Tilburg School of Humanities and Digital Sciences**,** Tilburg University, Tilburg, The Netherlands. The contributions of this author are as follows: Conceptualization, Writing–Review & Editing.

The correct citation is: Sadakata M, Weidema JL, Roncaglia-Denissen MPM, Honing H (2020) Parallel pitch processing in speech and melody: A study of the interference of musical melody on lexical pitch perception in speakers of Mandarin. PLoS ONE 15(3): e0229109. https://doi.org/10.1371/journal.pone.0229109

The correct Funding statement is as follows: JW, MPRD and HH were supported by a Horizon grant (317-70-010) of the Netherlands Organization for Scientific Research (NWO). HH was supported by a Distinguished Lorentz fellowship granted by the Lorentz Center for the Sciences and the Netherlands Institute for Advanced Study in the Humanities and Social Sciences (NIAS).

The correct Acknowledgements statement is as follows: The authors are very grateful to Loy Clements and Carlos Vaquero for their help in creating the code to generate the auditory stimuli. Additional appreciation to Loy Clements for his assistance in creating additional code for stimulus creation, EEG processing, and statistical analyses; and to Ya-Ping Hsiao and Yuan Yan for their assistance in recording and translating the Mandarin stimuli. The authors also express valued appreciation to Johan Tangerman for his help with data collection.
