# Supplementary figures and images for: Parallel pitch processing in speech and melody: A study of the interference of musical melody on lexical pitch perception in speakers of Mandarin
Source: PLoS One. 2020 Mar 4;15(3):e0229109. doi: 10.1371/journal.pone.0229109 (PMC7055904; doi:10.1371/journal.pone.0229109)

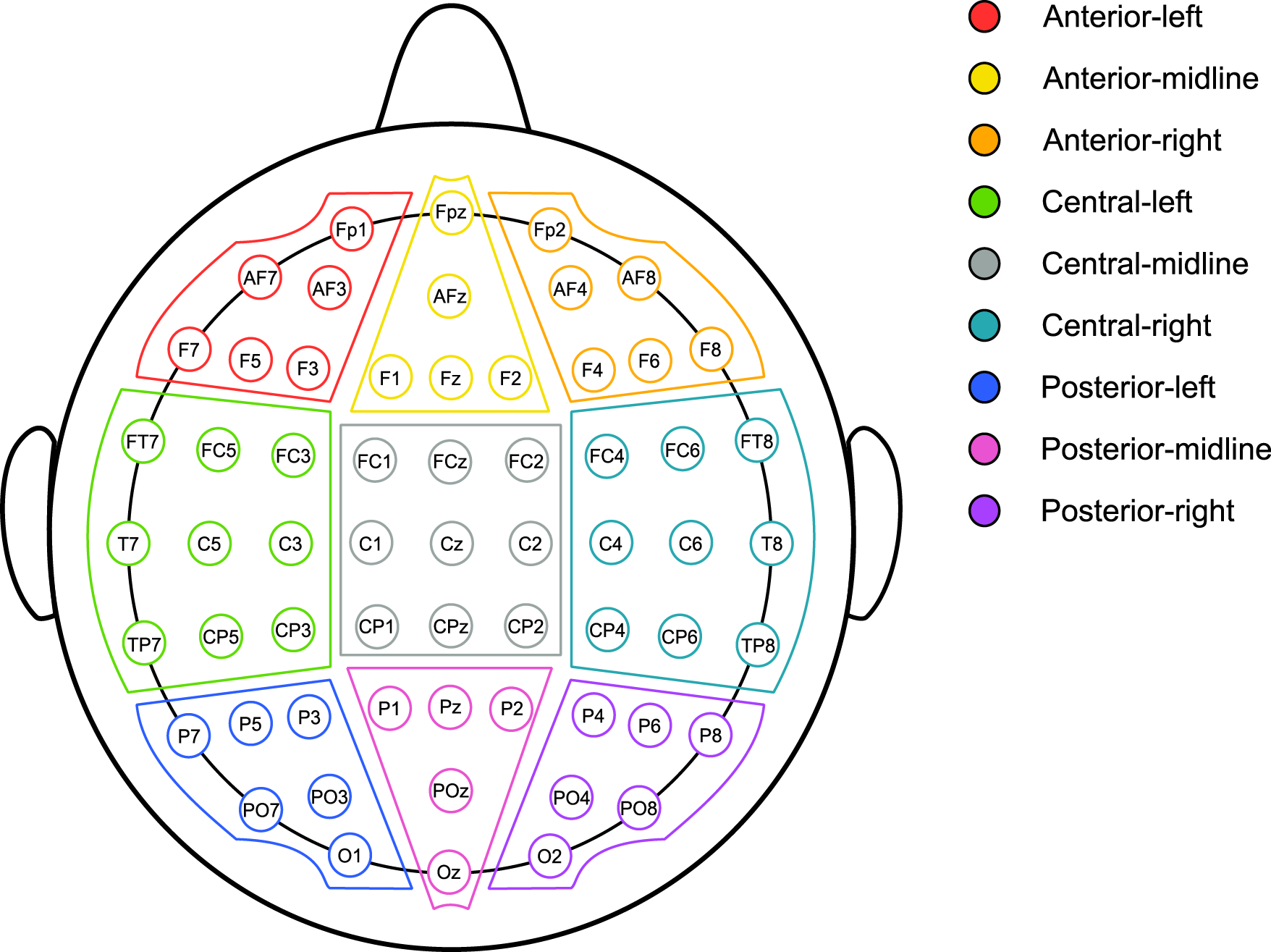

Supplement: S1 Fig — Regions are divided from top to bottom by Caudality (anterior, central, posterior) and from left to right by Laterality (left, midline, right). (PNG) [file pone.0229109.s001.png]
